# Longitudinal trajectories of depressive symptoms: the role of multimorbidity, mobility and subjective memory

**DOI:** 10.1186/s12877-023-03733-4

**Published:** 2023-01-12

**Authors:** Yiman Ji, Yiping Feng, Sijia Wu, Yutong Wu, Jiongjiong Wang, Xiangjuan Zhao, Yunxia Liu

**Affiliations:** 1 Department of Biostatistics, School of Public Health, Cheeloo College of Medicine, Shandong University, Jinan, 250012 Shandong China; 2 Institute for Medical Dataology, Cheeloo College of Medicine, Shandong University, Jinan, 250000 Shandong China; 3 Department of gynecology, Maternal and Child Health Care Hospital of Shandong Province, Jinan, 250014 Shandong China

**Keywords:** Trajectory, Depressive symptoms, Multimorbidity, Mobility limitation, Subjective memory impairment

## Abstract

**Background:**

The high prevalence of depression among older people in China places a heavy burden on the health system. Multimorbidity, mobility limitation and subjective memory impairment are found to be risk indicators for depression. However, most studies on this topic focused on depression at a single point in time, ignoring the dynamic changes in depressive symptoms and the relationship between the trajectories and these three conditions. Therefore, we aimed to identify distinct trajectories of depressive symptoms in older people and investigate their associations with multimorbidity, mobility limitation and subjective memory impairment.

**Methods:**

Data was drawn from China Health and Retirement Longitudinal Study conducted during 2011–2018. A total of 5196 participants who completed 4 visits, conducted every 2–3 years were included in this study. Group-based trajectory modeling was conducted to identify distinct trajectories of depressive symptoms z-scores. Multinomial logistic regression was used to investigate the relationships.

**Results:**

Four distinct trajectories of depressive symptoms z-scores were identified, labeled as persistently low symptoms (68.69%, *n* = 3569), increasing symptoms (12.14%, *n* = 631), decreasing symptoms (14.05%, *n* = 730) and persistently high symptoms (5.12%, *n* = 266). Participants with multimorbidity had unfavorable trajectories of depressive symptoms compared with those without multimorbidity, with adjusted odds ratios (95% *CIs*) of 1.40 (1.15, 1.70), 1.59 (1.33, 1.90) and 2.19 (1.65, 2.90) for the increasing symptoms, decreasing symptoms and persistently high symptoms, respectively. We also observed a similar trend among participants with mobility limitations. Compared with participants who had poor subjective memory, participants with excellent/very good/good subjective memory had a lower risk of developing unfavorable trajectories of depressive symptoms. The adjusted odds ratios (95% *CIs*) of the increasing symptoms, decreasing symptoms and persistently high symptoms were 0.54 (0.40, 0.72), 0.50 (0.38, 0.65) and 0.48 (0.31, 0.73), respectively.

**Conclusions:**

Multimorbidity, mobility limitation and subjective memory impairment were found to be potential risk factors for unfavorable depression trajectories.

**Supplementary Information:**

The online version contains supplementary material available at 10.1186/s12877-023-03733-4.

## Background

Major depression was listed as the third reason for the burden of disease worldwide by WHO and was projected to rank first by 2030 [1, 2]. The overall prevalence of depressive symptoms among older people in China is 20.0% [3]. Findings from systematic reviews including 124, 23 and 14 studies, respectively, have shown that depression is associated with a variety of common adverse outcomes such as coronary heart disease, diabetes and frailty [4–6]. Depression can significantly reduce the quality of life in older people. The high prevalence of depression among older people in China puts a heavy burden on the health system [7].

Quality of life, a multi-dimensional concept, is tightly related to the trajectories of depressive symptoms. Our research focused on physical condition, physical functioning and mental impairment operationalized as multimorbidity, mobility limitation and subjective memory impairment, which are common in older adults [8–11]. Previous studies have examined the relationship between these three conditions and depression [12–17]. A meta-analysis concluded that people with multimorbidity were more likely to suffer from depression than those without multimorbidity [12]. Stek et al. reported that depression in older people was strongly associated with mobility limitation [15]. Several studies also found that subjective memory impairment was a risk indicator for depression in previous studies [16, 17]. However, these studies focused on depression symptoms at a single time point. The dynamic trajectories of depressive symptoms and the relationship between the trajectories and these three conditions are still unclear.

We hypothesized that multimorbidity, mobility limitation and subjective memory impairment were associated with unfavorable depressive symptoms trajectories. Using repeated measures of depressive symptoms in 4 visits from the China Health and Retirement Longitudinal Study (CHARLS) during 2011–2018, the current study aims to identify distinct trajectories of depressive symptoms in older people, and investigate their associations with multimorbidity, mobility and subjective memory.

## Methods

### Study cohort

CHARLS is a population-based, prospective cohort study that aims to collect a set of high-quality micro-data representing families and individuals in China to analyze the population aging issues and to promote interdisciplinary research on aging [18]. The survey adopted a four-stage, stratified, cluster probability sampling process to sample 17,708 middle-aged and older individuals from 150 counties in 28 provinces. The first visit of CHARLS was launched in 2011 and participants subsequently completed 3 follow-up visits (wave 2 in 2013, wave 3 in 2015 and wave 4 in 2018).

We excluded individuals with missing information on depressive symptoms, mobility, subjective memory, multimorbidity and other covariates, as well as individuals who were younger than 45 years, or were lost to follow-up in waves 2–4. In addition, individuals with cognitive impairment (defined as cognitive scores < 6 [1.5 SD below its mean]) were also excluded [19, 20]. A total of 5196 subjects who completed 4 visits conducted every 2–3 years were included in this study. Fig. S1 in Additional file 2 shows the detailed population selection process. Table S1 in Additional file 1 presents the baseline characteristics of excluded respondents (*n* = 12,512) who were generally older, more likely to be women and unmarried, less likely to be smokers or drinkers, more likely to have a lower educational level and a lower level of household income.

### Measures

#### Depressive symptoms

The Center for Epidemiologic Studies Depression Scale (CESD) was used to assessed depressive symptoms, which had been validated previously in Chinese older adults [21]. There were 10 questions in total about the frequency they had experienced any of these 10 symptoms in the past week. Participants responded to these questions on a 4 point scale (0 = rarely; 1 = some days; 2 = occasionally; 3 = most of the time). The total score was calculated by summing the scores of the 10 questions, and ranged from 0 to 30 points. Depression symptoms were defined as CESD scores ≥10. Participants who had a complete assessment of depressive symptoms at each of the 4 visits were included. In this study, depressive symptom was used as a continuous variable based on the total score of CESD.

#### Multimorbidity

Participants were provided a list of 14 chronic conditions and were asked to select the conditions that their doctor diagnosed and that lasted at least half a year. Multimorbidity was defined as a binary variable where one group had one or none of the 14 chronic diseases and the other group had two or more chronic diseases.

#### Mobility

A scale of 9 items which has been proven to have good reliability was used to assess mobility, including running or jogging about 1 km; walking 1 km; walking 100 m; getting up from a chair after sitting for a long period; climbing several flights of stairs without resting; stooping, kneeling, or crouching; reaching or extending arms above shoulder level; lifting weights over 10 jin and picking up a small coin from a table [22]. Each item was coded as a dichotomous variable (0 = ‘no, I don’t have any difficulty’, 1=‘I have difficulty but can still do it’, ‘yes, I have difficulty and need help’ or ‘I cannot do it’). The summary score was obtained by adding the scores of the 9 items and transformed into disabled (summary scores ≥1) or not disabled (summary scores = 0) [22].

#### Subjective memory

Subjective memory was evaluated using a single item: ‘How would you rate your memory at the present time?’ with answer defined using 5 categories (excellent, very good, good, fair and poor) [23]. Responses for subjective memory impairment were recoded into 3 categories (excellent/very good/good, fair, and poor) for analytical purposes, considering that a small number of participants reported excellent, very good and good subjective memory (with percentage of 0.4, 6.4 and 15.2%).

#### Covariates

Covariates included age, gender, residence (rural and urban), marital status (married and unmarried), smoking status (smoker and non-smoker), drinking (drinker and non-drinker), educational level, household income and cognition scores. Smoking status was evaluated using two questions: “Have you ever chewed tobacco, smoked a pipe, smoked self-rolled cigarettes, or smoked cigarettes/cigars?” and “Do you still have the habit or have you totally quit?” Drinking was determined using a single item: “Did you ever drink alcoholic beverages in the past? How often?” Educational level was coded as 4 categories (< primary school, primary school, middle school and ≥ high school). Household income was recoded according to tertiles (low, medium and high). Cognitive function was assessed through two categories including episodic memory and mental intactness. Immediate word recall and delayed word recall were used to evaluate episodic memory (range 0–20). Telephone Interview of Cognitive Status (TICS) was used to measure mental intactness. The TICS consisted of serial subtraction of 7 from 100 (range 0–5), the date (month, day, and year), day of the week, season of the year (range 0–5), and intersecting pentagon copying test (range 0–1). The total score was calculated as the sum of the items mentioned above (range 0–31).

### Statistical methods

The raw depressive symptoms scores were adjusted for age by regression analyses, and the predicted depressive symptoms scores were transformed using the following equation to obtain the adjusted z-scores:

$$z=\frac{Y-\overline{Y\hbox{'}}}{RMSE}$$

where *Y* is the raw depressive symptoms score, *Y*^′^ is the predicted mean score of depressive symptoms, and *RMSE* is the root mean square error for the regression model. The transformed z-scores were used in subsequent analyses [19, 24].

Group-based trajectory modeling (GBTM), based on a censored normal distribution, was conducted to identify distinct trajectories of depressive symptoms z-scores. GBTM is a finite mixture modeling application that uses trajectory groups as a statistical device to identify distinctive clusters of trajectories across the population over time or age and profile the characteristics of individuals within the clusters [25, 26]. GBTM assumes that the distribution of population is discrete but there is no intra-class variation among individuals in the same cluster. To determine the optimal number of groups that can best represent the heterogeneity of developmental trajectories, we first fitted a single model with 1 group and then iteratively expanded to 5 groups as a function of follow-up time. Follow-up time and its higher-order terms (up to cubic terms) were included one by one for model building. The model selection was determined by the following criteria [27]: high mean posterior probabilities (> 0.7); greater membership in each trajectory group (≥ 5.0%); a reduction of Bayesian information criterion (BIC) of at least 20. Higher-order terms were removed from the model if they were not significant or did not improve the goodness-of-fit of the model.

To compare characteristics of multiple different trajectory groups, Mann-Whitney test and Kruskal-Wallis test were used for continuous variables, and *χ*^2^ test was used for categorical variables. Multinomial logistic regression model was used to investigate the associations between multimorbidity, mobility, subjective memory and the trajectories of depressive symptoms z-scores. Multimorbidity, mobility and subjective memory were entered into the multinomial logistic regression models together, and the odds ratios (*ORs*) and corresponding 95% confidence intervals (*CIs*) were reported. We added covariates sequentially into 3 models: unadjusted in model 1; in model 2, adjusted for baseline age, gender, region, education level, marital status, household income, smoking and alcohol drinking; and in model 3, additionally adjusted for baseline cognition scores.

In sensitivity analyses, multinomial logistic regression models were performed separately in women and men to examine the potential gender differences in the relationship between multimorbidity, subjective memory, mobility and the trajectories of depressive symptoms z-scores. In addition, participants who had been treated for depression during the follow-up were excluded. Trajectory groups were determined using a SAS macro (PROC TRAJ) and other statistical analyses were conducted using R 4.0.3. The statistical significance level was set at *P* < 0.05.

## Results

Table 1 summarizes the baseline characteristics by sex. The mean age was 56.3 years at baseline and 53.7% were men. Most of the sample were married (93.3%), lived in rural areas (77.2%) and had educational levels below high school (83.2%). The proportions of smokers (42.7%) and drinkers (44.9%) were roughly similar. The mean cognition and depression scores of all the participants were 15.5 and 9.5. 43.7, 35.8, 58.5 and 26.4% of participants reported depression symptoms, multimorbidity, mobility limitation and poor subjective memory, respectively. The male participants were older, more likely to be married, live in rural areas and have higher educational levels and higher household income than female participants. In addition, there were far more smokers and drinkers among male participants than female participants. In terms of the proportions of participants with multimorbidity, mobility limitation and poor subjective memory, it was significantly lower in male participants in comparison with female participants.

**Table 1:** Baseline characteristics of the study cohort from CHARLS stratified by sex

Characteristic	Total (*n* = 5196)	Male (*n* = 2788)	Female (*n* = 2408)	*P* value
Age, years	56.3 (7.7)	57.4 (7.8)	55.1 (7.4)	< 0.001
Rural, n (%)	4011 (77.2)	2228 (79.9)	1783 (74.0)	< 0.001
Married, n (%)	4847 (93.3)	2637 (94.6)	2210 (91.8)	< 0.001
Educational level, n (%)				< 0.001
< Primary school	1404 (27.0)	507 (18.2)	897 (37.3)	
Primary school	1366 (26.3)	775 (27.8)	591 (24.5)	
Middle school	1553 (29.9)	932 (33.4)	621 (25.8)	
≥ High school	873 (16.8)	574 (20.6)	299 (12.4)	
Household income, n (%)^a^				< 0.001
Low	1588 (30.6)	789 (28.3)	799 (33.2)	
Medium	1611 (31.0)	878 (31.5)	733 (30.4)	
High	1652 (31.8)	983 (35.3)	669 (27.8)	
Smoker, n (%)	2219 (42.7)	2046 (73.4)	173 (7.2)	< 0.001
Drinker, n (%)	2335 (44.9)	1950 (69.9)	385 (16.0)	< 0.001
Cognition scores	15.5 (4.4)	15.7 (4.2)	15.2 (4.5)	< 0.001
Depression scores	9.5 (4.6)	8.9 (4.3)	10.3 (4.8)	< 0.001
Depression symptoms, n (%)	2269 (43.7)	1060 (38.0)	1209 (50.2)	< 0.001
Multimorbidity, n (%)	1859 (35.8)	960 (34.4)	899 (37.3)	0.030
Mobility limitation, n (%)	3039 (58.5)	1403 (50.3)	1636 (67.9)	< 0.001
Subjective memory, n (%)				< 0.001
Poor	1371 (26.4)	621 (22.3)	750 (31.1)	
Fair	2677 (51.5)	1486 (53.3)	1191 (49.5)	
Excellent/very good/good	1148 (22.1)	681 (24.4)	467 (19.4)	

Data are means (SD), or n (%)

^a^: results of household income were calculated after removing missing value

*P* values: the differences between male group and female group

Table S2 in Additional file 1 presents detailed information about the results of the GBTM fitting process. The BIC was lower for the models with 5 or 6 trajectories (BIC = − 26,958.26/ -26,891.40). However, the average posterior probabilities were less than 0.7 for several of the trajectory groups. Finally, we selected a model of cubic parameters with 4 groups which had lower BIC, higher average posterior probabilities and group membership probability. In addition, we removed the nonsignificant cubic terms from the model.

**Fig. 1:**
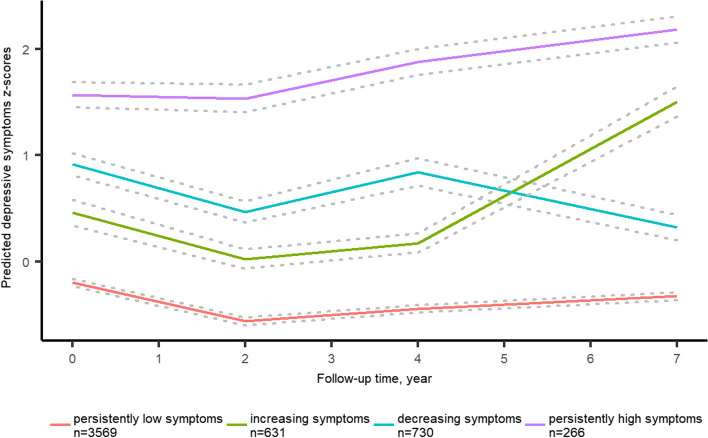
Trajectories of Depressive Symptoms from 2011 to 2018. The trajectories were shown in solid lines, and the 95% confidence intervals (CIs) were shown in dash line. See detailed information on the curve parameters in Table S3

Figure 1 shows the 4 distinct trajectories of depressive symptoms z-score, labeled as persistently low symptoms (68.69%, *n* = 3569), increasing symptoms (12.14%, *n* = 631), decreasing symptoms (14.05%, *n* = 730) and persistently high symptoms (5.12%, *n* = 266). Participants in the persistently low symptoms group had a low level of depressive symptoms throughout the follow-up, although it fluctuated slightly. The increasing symptoms group followed a quadratic trend. The level of depressive symptoms in this group was low at baseline and fluctuated slightly between visit 1 and visit 3. However, it rose sharply to a level higher than decreasing symptoms group during visit 3 and visit 4. In the decreasing symptoms group, the level of depressive symptoms was higher than that in the increasing symptoms at baseline, but gradually decreased to lower than that in the increasing symptoms group at visit 4. In regard to the persistently high symptoms group, the level of depressive symptoms was consistently significantly higher than that in the persistently low symptoms group throughout the follow-up. The trajectory parameters were all significantly different from 0 except for the cubic term in the increasing symptoms group and the linear term in the persistently high symptoms group. The curve parameters of the optimal model with 4 trajectory groups are shown in Table S3.

The baseline characteristics stratified by depression trajectory groups are shown in Table 2. The persistently high symptoms group had the highest proportion of participants with depression symptoms, multimorbidity, mobility limitation and poor subjective memory, followed by the decreasing symptoms and increasing symptoms, and those in the persistently low symptoms group were the lowest. In addition, significant differences (*P* < 0.001) among the 4 trajectory groups were observed in all included variables at baseline.

**Table 2:** Baseline characteristics of participants by trajectories of depressive symptoms

Characteristic	Persistently low symptoms	Increasing symptoms	Decreasing symptoms	Persistently high symptoms	*P* value
Age, years	56.6 (7.8)	55.6 (7.5)	55.8 (7.6)	56.3 (7.7)	0.005
Male, n (%)	2152 (60.3)	257 (40.7)	303 (41.5)	76 (28.6)	< 0.001
Rural, n (%)	2664 (74.6)	508 (80.5)	605 (82.9)	234 (88.0)	< 0.001
Married, n (%)	3351 (93.9)	586 (92.9)	681 (93.3)	229 (86.1)	< 0.001
Educational level, n (%)					< 0.001
< Primary school	839 (23.5)	213 (33.8)	231 (31.6)	121 (45.5)	
Primary school	947 (26.5)	160 (25.4)	182 (24.9)	77 (28.9)	
Middle school	1105 (31.0)	169 (26.8)	225 (30.8)	54 (20.3)	
≥ High school	678 (19.0)	89 (14.1)	92 (12.6)	14 (5.3)	
Household income, n (%)^a^					< 0.001
Low	983 (27.5)	229 (36.3)	260 (35.6)	116 (43.6)	
Medium	1121 (31.4)	171 (27.1)	236 (32.3)	83 (31.2)	
High	1237 (34.7)	179 (28.4)	185 (25.3)	51 (19.2)	
Smoker, n (%)	1645 (46.1)	217 (34.4)	277 (37.9)	80 (30.1)	< 0.001
Drinker, n (%)	1717 (48.1)	243 (38.5)	284 (38.9)	91 (34.2)	< 0.001
Cognition scores	15.9 (4.3)	14.9 (4.3)	14.6 (4.3)	13.6 (4.0)	< 0.001
Depression scores	7.9 (3.4)	11.2 (4.3)	13.7 (4.1)	16.5 (4.7)	< 0.001
Depression symptoms, n (%)	1007 (28.2)	401 (63.5)	610 (83.6)	251 (94.4)	< 0.001
Multimorbidity, n (%)	1110 (31.1)	260 (41.2)	338 (46.3)	151 (56.8)	< 0.001
Mobility limitation, n (%)	1842 (51.6)	423 (67.0)	557 (76.3)	217 (81.6)	< 0.001
Subjective memory, n (%)					< 0.001
Poor	736 (20.6)	208 (33.0)	292 (40.0)	135 (50.8)	
Fair	1927 (54.0)	327 (51.8)	329 (45.1)	94 (35.3)	
Excellent/very good/good	906 (25.4)	96 (15.2)	109 (14.9)	37 (13.9)	

Data are means (SD), or n (%)

^a^: results of household income were calculated after removing missing value

*P* values: the differences between persistently low symptoms, increasing symptoms, decreasing symptoms and persistently high symptoms

Table 3 presents the *ORs* and 95% *CIs* for the associations of multimorbidity, subjective memory, mobility and the trajectories of depressive symptoms z-scores. Participants with multimorbidity had worse depressive symptoms trajectories compared with participants without multimorbidity, with unadjusted *ORs* (95% *CIs*) for the increasing symptoms, decreasing symptoms and persistently high symptoms group of 1.28 (1.06, 1.53), 1.42 (1.20, 1.68) and 2.01 (1.56, 2.63), respectively. After adjusting for baseline age, gender, region, education level, marital status, household income, smoking and alcohol drinking in model 2, the *ORs* (95% *CIs*) for the increasing symptoms, decreasing symptoms and persistently high symptoms group were 1.38 (1.14, 1.68), 1.57 (1.31, 1.88) and 2.15 (1.62, 2.84), respectively. After additional adjustment for baseline cognition score in model 3, the *ORs* (95% *CIs*) were 1.40 (1.15, 1.70), 1.59 (1.33, 1.90) and 2.19 (1.65, 2.90), respectively. We also observed a similar trend among people who reported mobility limitations. Compared with participants who had poor subjective memory, participants with better subjective memory had a lower risk of developing worse depressive symptoms trajectories. Among participants with fair subjective memory, the unadjusted *ORs* (95% *CIs*) for the increasing symptoms, decreasing symptoms and persistently high symptoms group were 0.66 (0.54, 0.80), 0.50 (0.42, 0.60) and 0.33 (0.25, 0.43), respectively. With regard to participants with excellent/very good/good subjective memory, the unadjusted *ORs* (95% *CIs*) for the increasing symptoms, decreasing symptoms and persistently high symptoms group were 0.46 (0.35, 0.60), 0.42 (0.33, 0.54) and 0.35 (0.24, 0.52), respectively. Moreover, the results were still robust after adjusting for the covariates mentioned above in model 2 and model 3.

**Table 3:** Multinomial logistic regression analysis between multimorbidity, mobility and subjective memory and depression trajectories

	Increasing symptoms (vs. Persistently low symptoms)		Decreasing symptoms (vs. Persistently low symptoms)		Persistently high symptoms (vs. Persistently low symptoms)	
	OR (95%CI)	*P* value	OR (95%CI)	*P* value	OR (95%CI)	*P* value
**Model 1**						
Multimorbidity (ref: No)	1.28 (1.06, 1.53)	0.008	1.42 (1.20, 1.68)	< 0.001	2.01 (1.56, 2.63)	< 0.001
Mobility (ref: Not disabled)	1.60 (1.33, 1.93)	< 0.001	2.41 (1.99, 2.92)	< 0.001	2.88 (2.07, 4.00)	< 0.001
Subjective memory (ref: Poor)						
Fair	0.66 (0.54, 0.80)	< 0.001	0.50 (0.42, 0.60)	< 0.001	0.33 (0.25, 0.43)	< 0.001
Excellent/very good/good	0.46 (0.35, 0.60)	< 0.001	0.42 (0.33, 0.54)	< 0.001	0.35 (0.24, 0.52)	< 0.001
**Model 2**						
Multimorbidity (ref: No)	1.38 (1.14, 1.68)	0.001	1.57 (1.31, 1.88)	< 0.001	2.15 (1.62, 2.84)	< 0.001
Mobility (ref: Not disabled)	1.45 (1.19, 1.78)	< 0.001	2.27 (1.85, 2.79)	< 0.001	2.26 (1.59, 3.22)	< 0.001
Subjective memory (ref: Poor)						
Fair	0.69 (0.56, 0.85)	< 0.001	0.52 (0.43, 0.64)	< 0.001	0.39 (0.29, 0.53)	< 0.001
Excellent/very good/good	0.51 (0.38, 0.67)	< 0.001	0.47 (0.36, 0.61)	< 0.001	0.43 (0.28, 0.65)	< 0.001
**Model 3**						
Multimorbidity (ref: No)	1.40 (1.15, 1.70)	0.001	1.59 (1.33, 1.90)	< 0.001	2.19 (1.65, 2.90)	< 0.001
Mobility (ref: Not disabled)	1.43 (1.17, 1.75)	0.001	2.23 (1.81, 2.74)	< 0.001	2.20 (1.54, 3.13)	< 0.001
Subjective memory (ref: Poor)						
Fair	0.73 (0.59, 0.90)	0.004	0.56 (0.46, 0.68)	< 0.001	0.44 (0.32, 0.59)	< 0.001
Excellent/very good/good	0.54 (0.40, 0.72)	< 0.001	0.50 (0.38, 0.65)	< 0.001	0.48 (0.31, 0.73)	< 0.001

Model 1: Unadjusted for any covariates

Model 2: Adjusted for baseline age, gender, region, education level, marital status, household income, smoking and alcohol drinking

Model 3: Adjusted for baseline age, gender, region, education level, marital status, household income, smoking, alcohol drinking and cognition scores

The results of sensitivity analyses were basically consistent with the main analyses. In gender-specific analyses, there was similar trend to the whole population of the relationship between multimorbidity, mobility limitation, subjective memory impairment and trajectories of depressive symptoms z-scores. However, the *OR* of the increasing symptoms group for women with multimorbidity was not significantly different from 1. Similarly, the *ORs* of the “increasing symptoms” group for men with fair subjective memory and the *ORs* of the “persistently high symptoms” group for men with excellent/very good/good subjective memory in model 2 and model 3 were not statistically significant (Table S4 and Table S5 in  Additional file 1). The results were largely consistent with the main analyses after 32 participants who had been treated for depression were excluded. (Table S6 in Additional file 1).

## Discussion

Using a nationally prospective cohort from CHARLS, we characterized the trajectories of depressive symptoms z-scores in older people and explored the associations between multimorbidity, mobility, subjective memory and the trajectories of depressive symptoms z-scores. Four distinct trajectories of depressive symptoms were identified characterized by persistently low symptoms, increasing symptoms, decreasing symptoms and persistently high symptoms. Participants with multimorbidity or mobility limitation had worse depression trajectories, while participants with better subjective memory had a lower risk of developing worse depressive symptoms trajectories. This study provides new insights into the potential risk factors for depressive symptoms in older people, and highlights the importance of comprehensively assessing the risk of depression in older people through the physical condition, mental impairment, and physical functioning.

There are several studies focusing on identifying diverse depression z-scores trajectories in older people [28–30]. Kaup et al. identified 3 depressive symptoms trajectories comprising consistently minimal symptoms, moderate and increasing symptoms, and high and increasing symptoms [28]. Holmes et al. reported 2 predominant trajectories of depressive symptoms including a chronically elevated trajectory and a low, stable symptom trajectory [29]. The reason why the results of this study are inconsistent with the above two studies is that the study population is different in race and follow-up years. The data used in this study were derived from a latest cohort of Chinese, which makes this study more generalizable in Chinese population. Li et al. observed 4 trajectories of depressive symptoms characterized by low symptoms, decreasing symptoms, increasing symptoms, and high symptoms which were similar to the present study [30]. But our study added the latest data from visit 4 with a longer follow-up period, and the trajectory analysis was more stable because only participants with data of all 4 visits could be included.

The results of multinomial logistic regression showed that older adults with multimorbidity had worse trajectories of depressive symptoms compared with those without multimorbidity. Few studies focused on the association between multimorbidity and depression trajectories [31, 32]. Hsu et al. aimed to examine the group-based trajectories of depressive symptoms in older people and to identify risk factors for these trajectories [31]. They found that for older men and women, higher-level trajectories of depressive symptoms were related to more chronic diseases. Additionally, they further explored the relationship between comorbidity and depressive symptom trajectories in the same population and concluded that all different patterns of chronic disease were related to higher depressive symptoms which was consistent with our study [32]. The underlying mechanisms of the relationship between multimorbidity and depression trajectories are unclear. Hypercortisolemia, which occurs during chronic diseases, is associated with increased amygdala activity and damage to the hippocampus, thereby increasing susceptibility to depression [33]. Moreover, the aversive symptoms and decreased quality of life associated with chronic diseases, as well as the indirect pathophysiological effects of these diseases on the brain through increased cytokine levels or other inflammatory factors, may lead to major depression [34].

In addition, the depressive symptoms trajectories of older people with mobility limitation were worse than those of older people without mobility limitation. This finding extends the existing literature on the relationship between mobility limitation and trajectories of depressive symptoms [35, 36]. Xiang et al. examined the trajectories of depressive symptoms in older adults and their associated factors [35]. They found mobility limitation was unique risk factor for “increasing” trajectory, rather than “declining” trajectory and “persistently high” trajectory. Brett et al. investigated the longitudinal course of depressive symptom and the impact of physical function and drew the conclusion that decline of physical function predicted the trajectory of increased depressive symptom [36]. However, since the study population was former American football players, the conclusions were not generalizable. Mobility limitation, defined as one of the six key dimensions of psychological wellness, reflects the individual’s ability to choose or create an environment appropriate to their mental condition [37]. Older adults with mobility limitation have poor psychological wellness leading to worse trajectories of depressive symptoms.

Furthermore, the association between subjective memory impairment and depressive symptoms is complex and may be bidirectional [16, 17, 38]. This study proved that subjective memory impairment may be a potential risk factor for depressive symptoms by examining the dynamic trajectories of depressive symptoms z-scores. Several potential explanations support these observed associations. First, participants with mild cognitive impairment suffer from forgetfulness and fear of developing dementia, which could trigger depression, so depression may be a “reactive” response to mild cognitive impairment [39]. Second, previous studies have shown that depressive symptoms predict the transition from mild cognitive impairment to all-cause dementia, meaning that depression is an intermediate stage between mild cognitive impairment and dementia [39, 40]. Third, participants without severe cognitive problems reported subjective memory impairment that may be a manifestation of depression [41].

The current study is one of the first studies to focus on the trajectories of depressive symptoms among older Chinese adults and the associations of multimorbidity, mobility limitation and subjective memory impairment with the trajectories. Recently, Yan et al. analyzed the trajectories of depression and their associations with multimorbidity and disability [42]. But our study has the added advantage of using advanced statistical models (GBTM) to fit the distinct trajectories of depressive symptoms. Two main advantages of the GBTM are the ability to identify distinct developmental processes that are not easily identified using prior classification rules, and the capacity for distinguishing between chance variations and real differences among individuals [25]. Xiang et al. examined depression trajectories and explored the relationship between mobility difficulties, cognitive impairment, chronic disease count and depression trajectories in a representative sample of older adults in the United States [43]. However, they did not find that mobility difficulties were associated with a higher risk of being on the “decreasing” trajectory and “persistently moderate/high” compared with the “never” trajectory. In addition, they reported that cognitive impairment was associated with a lower risk of being on the “increasing” and “decreasing” trajectories which was inconsistent with our study. The discrepancies between the results may be due to different screening conditions of the study population. Only participants with all of the 4 visits were included in our study, and the data can reflect relatively complete trajectory change information and increase the probability of correct classification. The correct classification of trajectories may affect the result of multinomial logistic regression.

### Strengths and limitations

This study has several strengths. Firstly, the data set was drawn from a nationally representative cohort with comprehensive and rigorous measurements of risk factors, which gave us the opportunity to adjust for more covariates. Secondly, with the help of GBTM, we were able to characterize distinct groups of participants with similar levels and change patterns of depression scores. Thirdly, two sensitivity analyses were conducted to explore whether there were gender differences in the results of multinomial logistic regression model and to demonstrate the robustness of the results. There are also several potential limitations. First, a large proportion of participants were excluded due to loss of follow-up or missing information as shown in Fig. S1, and the differences between the included and excluded groups could lead to selection bias. Second, the assessments of depressive symptoms, multimorbidity, subjective memory and mobility were based on self-reports through questionnaires. Recall bias and the underestimation of research results were still inevitable. Third, although we had adjusted for several covariates obtained from this large observational cohort, we still could not make causal inferences because residual confounding could not be completely eliminated.

## Conclusions

In summary, this study distinguished 4 distinct trajectories of depressive symptoms z-scores in older people. Multimorbidity, mobility limitation and subjective memory impairment were found to be potential risk factors for worse trajectories of depressive symptoms. This study provides new insights into the potential risk factors for depressive symptoms in older people, and highlights the importance of evaluating the risk of depression in older people comprehensively through physical condition, physical functioning and mental impairment. The risk factors identified in this study could be used as screening and monitoring variables for depression and as criteria for selecting population for early intervention.

## Supplementary Information

**Additional file 1: Table S1.** Baseline characteristics of participants included and excluded. **Table S2.** Group-based trajectory modeling results of model fitting process. **Table S3.** Parameter estimates for the best fitting model. **Table S4.** Multinomial logistic regression analysis between multimorbidity, mobility and subjective memory and depression trajectories in women. **Table S5.** Multinomial logistic regression analysis between multimorbidity, mobility and subjective memory and depression trajectories in men. **Table S6.** Multinomial logistic regression analysis between multimorbidity, mobility and subjective memory and depression trajectories excluding participants who had antidepressant use.

**Additional file 2:** **Fig. S1.** Flow chart of the study population selection.

## Data Availability

The data from this analysis are publicly available from the China Health and Retirement Longitudinal Study (CHARLS) website: http://charls.pku.edu.cn/en.

## Abbreviations

CHARLS: China Health and Retirement Longitudinal Study
CESD: Center for Epidemiologic Studies Depression Scale
GBTM: Group-based trajectory modeling
BIC: Bayesian information criterion
OR: Odds ratio
CI: Confidence interval.
