# Supplementary material for: Longitudinal trajectories of depressive symptoms: the role of multimorbidity, mobility and subjective memory
Source: BMC Geriatr. 2023 Jan 12;23:22. doi: 10.1186/s12877-023-03733-4 (PMC9837987; doi:10.1186/s12877-023-03733-4)
Supplement: Supplementary file 1 — Additional file 1: Table S1. Baseline characteristics of participants included and excluded. Table S2. Group-based trajectory modeling results of model fitting process. Table S3. Parameter estimates for the best fitting model. Table S4. Multinomial logistic regression analysis between multimorbidity, mobility and subjective memory and depression trajectories in women. Table S5. Multinomial logistic regression analysis between multimorbidity, mobility and subjective memory and depression trajectories in men. Table S6. Multinomial logistic regression analysis between multimorbidity, mobility and subjective memory and depression trajectories excluding participants who had antidepressant use. [file 12877_2023_3733_MOESM1_ESM.docx]

**Table S1.** Baseline characteristics of participants included and excluded

| Characteristic | Excluded | Included | *P* value |
| --- | --- | --- | --- |
| *N* | 12512 | 5196 |  |
| Age, years | 60.1 (10.8) | 56.3 (7.7) | <0.001 |
| Male, n (%) | 5683 (45.4) | 2788 (53.7) | <0.001 |
| Rural, n (%) | 9493 (75.9) | 4011 (77.2) | 0.106 |
| Married, n (%) | 10570 (84.5) | 4847 (93.3) | <0.001 |
| Educational level, n (%) | | | <0.001 |
| < Primary school | 6539 (52.3) | 1404 (27.0) |  |
| Primary school | 2446 (19.5) | 1366 (26.3) |  |
| Middle school | 2099 (16.8) | 1553 (29.9) |  |
| ≥High school | 1373 (11.0) | 873 (16.8) |  |
| Household income, n (%)* |  |  | <0.001 |
| Low | 4549 (36.4) | 1588 (30.6) |  |
| Medium | 3155 (25.2) | 1611 (31.0) |  |
| High | 3406 (27.2) | 1652 (31.8) |  |
| Smoker, n (%) | 4744 (37.9) | 2219 (42.7) | <0.001 |
| Drinker, n (%) | 4875 (39.0) | 2335 (44.9) | <0.001 |
| Cognition scores | 10.1 (6.8) | 15.5 (4.4) | <0.001 |
| Depression scores | 10.3 (5.0) | 9.5 (4.6) | <0.001 |
| Depression symptoms, n (%) | 5016 (40.1) | 2269 (43.7) |  |
| Multimorbidity, n (%) | 4536 (36.3) | 1859 (35.8) | <0.001 |
| Mobility, n (%) | 6877 (55.0) | 3039 (58.5) | <0.001 |
| Subjective memory, n (%) |  |  | <0.001 |
| Poor | 4277 (34.2) | 1371 (26.4) |  |
| Fair | 4524 (36.2) | 2677 (51.5) |  |
| Excellent/very good/good | 2046 (16.4) | 1148 (22.1) |  |

Data are means (SD), or n (%)

Results were calculated after removing missing value

*P* value: the differences between the excluded and included

**Table S2.** Group-based trajectory modeling results of model fitting process

| NO.latent class | Polynomial degree | BIC | AIC | %Participants per class | Mean posterior probabilities |
| --- | --- | --- | --- | --- | --- |
| 1 | Linear | -29507.03 | -29497.20 | 100 | NA |
|  | Quadratic | -29325.24 | -29312.12 | 100 | NA |
|  | Cubic | -29264.35 | -29247.96 | 100 | NA |
| 2 | Linear | -27800.36 | -27780.69 | 79.54/20.46 | 96.02/88.58 |
|  | Quadratic | -27542.38 | -27516.16 | 79.18/20.82 | 96.01/89.03 |
|  | Cubic | -27455.67 | -27422.89 | 79.04/20.96 | 96.02/89.08 |
| 3 | Linear | -27548.86 | -27519.36 | 67.96/26.94/5.10 | 92.30/81.32/87.22 |
|  | Quadratic | -27261.20 | -27221.87 | 67.28/27.47/5.24 | 92.45/81.69/88.50 |
|  | Cubic | -27167.98 | -27118.81 | 67.19/27.58/5.23 | 92.55/82.03/88.66 |
| 4 | Linear | -27561.69 | -27522.36 | 28.24/39.72/26.94/5.10 | -/55.04/76.82/87.22 |
|  | Quadratic | -27131.47 | -27079.02 | 67.12/13.85/14.00/5.02 | 92.53/72.66/72.22/85.85 |
|  | Cubic | -27028.52 | -26962.97 | 66.64/13.75/14.50/5.11 | 92.60/72.23/73.13/86.78 |
| 5 | Linear | -27574.52 | -27525.36 | 22.02/20.52/25.42/26.94/5.10 | -/-/35.68/75.90/87.10 |
|  | Quadratic | -27070.89 | -27005.33 | 7.60/65.96/11.56/9.81/5.08 | 65.62/92.43/70.21/69.31/85.36 |
|  | Cubic | -26958.26 | -26876.32 | 65.23/7.35/12.60/9.44/5.38 | 92.51/67.21/71.66/68.89/86.27 |
| 6 | Linear | -27479.47 | -27420.47 | 44.23/2.85/21.42/15.32/12.45/3.73 | 61.16/62.74/51.17/60.37/66.32/82.41 |
|  | Quadratic | -27064.04 | -26985.37 | 64.66/11.76/8.34/7.26/2.84/5.13 | 91.61/60.93/65.19/66.29/65.92/84.57 |
|  | Cubic | -26891.40 | -26793.06 | 64.26/6.33/7.11/6.34/10.72/5.34 | 92.73/66.39/66.98/68.39/70.72/87.23 |
|  | **3,2,3,3** | **-27024.76** | **-26962.48** | **66.55/13.30/15.01/5.14** | **92.59/72.55/72.76/86.76** |

No.Latent class: latent class number of the model

BIC: Bayesian information criterion

AIC: Akaike information criterion

%Participants per class: proportion of participants per class

The best fitting model is highlighted in bold characters

**Table S3.** Parameter estimates for the best fitting model

| Trajectory group | Parameter | Maximum likelihood estimates | | | |
| --- | --- | --- | --- | --- | --- |
|  |  | *Est.* | *SE* | *Z* value | *P* value |
| Class1: Persistently low symptoms | Intercept | -0.198 | 0.015 | -13.039 | <0.001 |
|  | Linear | -0.389 | 0.024 | -16.045 | <0.001 |
|  | Quadratic | 0.119 | 0.010 | 12.243 | <0.001 |
|  | Cubic | -0.009 | 0.001 | -10.071 | <0.001 |
| Class 2: Increasing symptoms | Intercept | 0.460 | 0.060 | 7.624 | <0.001 |
|  | Linear | -0.366 | 0.035 | -10.366 | <0.001 |
|  | Quadratic | 0.074 | 0.005 | 14.862 | <0.001 |
| Class 3: Decreasing symptoms | Intercept | 0.913 | 0.052 | 17.434 | <0.001 |
|  | Linear | -0.634 | 0.074 | -8.562 | <0.001 |
|  | Quadratic | 0.255 | 0.030 | 8.455 | <0.001 |
|  | Cubic | -0.025 | 0.003 | -8.270 | <0.001 |
| Class 4: Persistently high symptoms | Intercept | 1.566 | 0.060 | 26.225 | <0.001 |
|  | Linear | -0.181 | 0.099 | -1.835 | 0.067 |
|  | Quadratic | 0.100 | 0.040 | 2.507 | 0.012 |
|  | Cubic | -0.009 | 0.004 | -2.285 | 0.022 |

*Est.*: parameter estimate

*SE*: standard error of parameter estimate

**Table S4.** Multinomial logistic regression analysis between multimorbidity, mobility and subjective memory and depression trajectories in women

|  | Increasing symptoms  (vs. Persistently low symptoms) | | Decreasing symptoms  (vs. Persistently low symptoms) | | Persistently high symptoms  (vs. Persistently low symptoms) | |
| --- | --- | --- | --- | --- | --- | --- |
|  | OR (95%CI) | *P* value | OR (95%CI) | *P* value | OR (95%CI) | *P* value |
| **Model 1** | | | | | | |
| Multimorbidity (ref: No) | 1.15 (0.90, 1.47) | 0.272 | 1.55 (1.23, 1.95) | <0.001 | 2.08 (1.51, 2.86) | <0.001 |
| Mobility (ref: Not disabled) | 1.45 (1.11, 1.88) | 0.006 | 2.18 (1.66, 2.88) | <0.001 | 2.36 (1.55, 3.59) | <0.001 |
| Subjective memory (ref: Poor) |  |  |  |  |  |  |
| Fair | 0.64 (0.49, 0.83) | 0.001 | 0.57 (0.44, 0.72) | <0.001 | 0.36 (0.26, 0.51) | <0.001 |
| Excellent/very good/good | 0.44 (0.31, 0.63) | <0.001 | 0.37 (0.26, 0.53) | <0.001 | 0.31 (0.19, 0.51) | <0.001 |
| **Model 2** | | | | | | |
| Multimorbidity (ref: No) | 1.20 (0.92, 1.56) | 0.173 | 1.68 (1.31, 2.15) | <0.001 | 1.99 (1.41, 2.82) | <0.001 |
| Mobility (ref: Not disabled) | 1.52 (1.15, 2.01) | 0.004 | 2.42 (1.79, 3.25) | <0.001 | 2.28 (1.46, 3.58) | <0.001 |
| Subjective memory (ref: Poor) |  |  |  |  |  |  |
| Fair | 0.65 (0.49, 0.86) | 0.002 | 0.58 (0.44, 0.75) | <0.001 | 0.42 (0.29, 0.61) | <0.001 |
| Excellent/very good/good | 0.48 (0.33, 0.71) | <0.001 | 0.41 (0.28, 0.60) | <0.001 | 0.37 (0.21, 0.63) | <0.001 |
| **Model 3** | | | | | | |
| Multimorbidity (ref: No) | 1.21 (0.93, 1.57) | 0.163 | 1.69 (1.32, 2.16) | <0.001 | 2.01 (1.42, 2.85) | <0.001 |
| Mobility (ref: Not disabled) | 1.50 (1.13, 1.98) | 0.005 | 2.37 (1.76, 3.19) | <0.001 | 2.21 (1.41, 3.47) | 0.001 |
| Subjective memory (ref: Poor) |  |  |  |  |  |  |
| Fair | 0.69 (0.52, 0.91) | 0.009 | 0.62 (0.47, 0.81) | 0.001 | 0.47 (0.32, 0.68) | <0.001 |
| Excellent/very good/good | 0.51 (0.34, 0.75) | 0.001 | 0.44 (0.30, 0.65) | <0.001 | 0.41 (0.23, 0.71) | 0.001 |

Model 1: Unadjusted for any covariates

Model 2: Adjusted for baseline age, region, education level, marital status, household income, smoking and alcohol drinking

Model 3: Adjusted for baseline age, region, education level, marital status, household income, smoking, alcohol drinking and cognition scores

**Table S5.** Multinomial logistic regression analysis between multimorbidity, mobility and subjective memory and depression trajectories in men

|  | Increasing symptoms  (vs. Persistently low symptoms) | | Decreasing symptoms  (vs. Persistently low symptoms) | | Persistently high symptoms  (vs. Persistently low symptoms) | |
| --- | --- | --- | --- | --- | --- | --- |
|  | OR (95%CI) | *P* value | OR (95%CI) | *P* value | OR (95%CI) | *P* value |
| **Model 1** | | | | | | |
| Multimorbidity (ref: No) | 1.59 (1.21, 2.09) | 0.001 | 1.32 (1.02, 1.71) | 0.032 | 2.20 (1.35, 3.57) | 0.001 |
| Mobility (ref: Not disabled) | 1.42 (1.07, 1.87) | 0.014 | 2.24 (1.71, 2.95) | <0.001 | 2.57 (1.47, 4.47) | 0.001 |
| Subjective memory (ref: Poor) |  |  |  |  |  |  |
| Fair | 0.77 (0.56, 1.05) | 0.095 | 0.46 (0.35, 0.61) | <0.001 | 0.31 (0.19, 0.53) | <0.001 |
| Excellent/very good/good | 0.52 (0.35, 0.79) | 0.002 | 0.50 (0.35, 0.72) | <0.001 | 0.49 (0.26, 0.94) | 0.031 |
| **Model 2** | | | | | | |
| Multimorbidity (ref: No) | 1.67 (1.25, 2.22) | <0.001 | 1.43 (1.09, 1.87) | 0.009 | 2.39 (1.45, 3.95) | 0.001 |
| Mobility (ref: Not disabled) | 1.40 (1.04, 1.89) | 0.026 | 2.24 (1.68, 2.99) | <0.001 | 2.39 (1.34, 4.28) | 0.003 |
| Subjective memory (ref: Poor) |  |  |  |  |  |  |
| Fair | 0.75 (0.54, 1.04) | 0.082 | 0.46 (0.34, 0.61) | <0.001 | 0.35 (0.21, 0.61) | <0.001 |
| Excellent/very good/good | 0.53 (0.34, 0.82) | 0.004 | 0.52 (0.35, 0.75) | <0.001 | 0.53 (0.27, 1.05) | 0.070 |
| **Model 3** | | | | | | |
| Multimorbidity (ref: No) | 1.70 (1.27, 2.27) | <0.001 | 1.45 (1.11, 1.91) | 0.007 | 2.48 (1.50, 4.10) | <0.001 |
| Mobility (ref: Not disabled) | 1.38 (1.02, 1.86) | 0.034 | 2.20 (1.65, 2.94) | <0.001 | 2.36 (1.32, 4.22) | 0.004 |
| Subjective memory (ref: Poor) |  |  |  |  |  |  |
| Fair | 0.80 (0.57, 1.11) | 0.177 | 0.48 (0.36, 0.65) | <0.001 | 0.39 (0.23, 0.68) | 0.001 |
| Excellent/very good/good | 0.57 (0.36, 0.88) | 0.011 | 0.55 (0.38, 0.80) | 0.002 | 0.61 (0.31, 1.22) | 0.165 |

Model 1: Unadjusted for any covariates

Model 2: Adjusted for baseline age, region, education level, marital status, household income, smoking and alcohol drinking

Model 3: Adjusted for baseline age, region, education level, marital status, household income, smoking, alcohol drinking and cognition scores

**Table S6.** Multinomial logistic regression analysis between multimorbidity, mobility and subjective memory and depression trajectories excluding participants who had antidepressant use

|  | Increasing symptoms  (vs. Persistently low symptoms) | | Decreasing symptoms  (vs. Persistently low symptoms) | | Persistently high symptoms  (vs. Persistently low symptoms) | |
| --- | --- | --- | --- | --- | --- | --- |
|  | OR (95%CI) | *P* value | OR (95%CI) | *P* value | OR (95%CI) | *P* value |
| **Model 1** | | | | | | |
| Multimorbidity (ref: No) | 1.28 (1.06, 1.53) | 0.009 | 1.39 (1.18, 1.65) | <0.001 | 1.99 (1.53, 2.59) | <0.001 |
| Mobility (ref: Not disabled) | 1.61 (1.33, 1.94) | <0.001 | 2.44 (2.02, 2.96) | <0.001 | 2.94 (2.11, 4.10) | <0.001 |
| Subjective memory (ref: Poor) |  |  |  |  |  |  |
| Fair | 0.66 (0.54, 0.80) | <0.001 | 0.50 (0.41, 0.60) | <0.001 | 0.33 (025, 0.44) | <0.001 |
| Excellent/very good/good | 0.45 (0.35, 0.59) | <0.001 | 0.42 (0.33, 0.54) | <0.001 | 0.34 (0.23, 0.51) | <0.001 |
| **Model 2** | | | | | | |
| Multimorbidity (ref: No) | 1.38 (1.13, 1.67) | 0.001 | 1.54 (1.28, 1.84) | <0.001 | 2.11 (1.59, 2.79) | <0.001 |
| Mobility (ref: Not disabled) | 1.54 (1.26, 1.89) | <0.001 | 2.46 (2.00, 3.01) | <0.001 | 2.53 (1.78, 3.61) | <0.001 |
| Subjective memory (ref: Poor) |  |  |  |  |  |  |
| Fair | 0.68 (0.55, 0.84) | <0.001 | 0.51 (0.42, 0.62) | <0.001 | 0.39 (0.29, 0.52) | <0.001 |
| Excellent/very good/good | 0.49 (0.37, 0.65) | <0.001 | 0.46 (0.35, 0.60) | <0.001 | 0.41 (0.27, 0.52) | <0.001 |
| **Model 3** | | | | | | |
| Multimorbidity (ref: No) | 1.39 (1.14, 1.69) | 0.001 | 1.56 (1.30, 1.86) | <0.001 | 2.15 (1.62, 2.84) | <0.001 |
| Mobility (ref: Not disabled) | 1.52 (1.24, 1.86) | <0.001 | 2.42 (1.97, 2.97) | <0.001 | 2.47 (1.73, 3.52) | <0.001 |
| Subjective memory (ref: Poor) |  |  |  |  |  |  |
| Fair | 0.72 (0.58, 0.89) | 0.002 | 0.54 (0.44, 0.66) | <0.001 | 0.43 (0.32, 0.58) | <0.001 |
| Excellent/very good/good | 0.52 (0.39, 0.70) | <0.001 | 0.49 (0.37, 0.64) | <0.001 | 0.45 (0.30, 0.70) | <0.001 |

Model 1: Unadjusted for any covariates

Model 2: Adjusted for baseline age, gender, region, education level, marital status, household income, smoking and alcohol drinking

Model 3: Adjusted for baseline age, gender, region, education level, marital status, household income, smoking, alcohol drinking and cognition scores
