# Supplementary figures and images for: Longitudinal trajectories of depressive symptoms: the role of multimorbidity, mobility and subjective memory
Source: BMC Geriatr. 2023 Jan 12;23:22. doi: 10.1186/s12877-023-03733-4 (PMC9837987; doi:10.1186/s12877-023-03733-4)

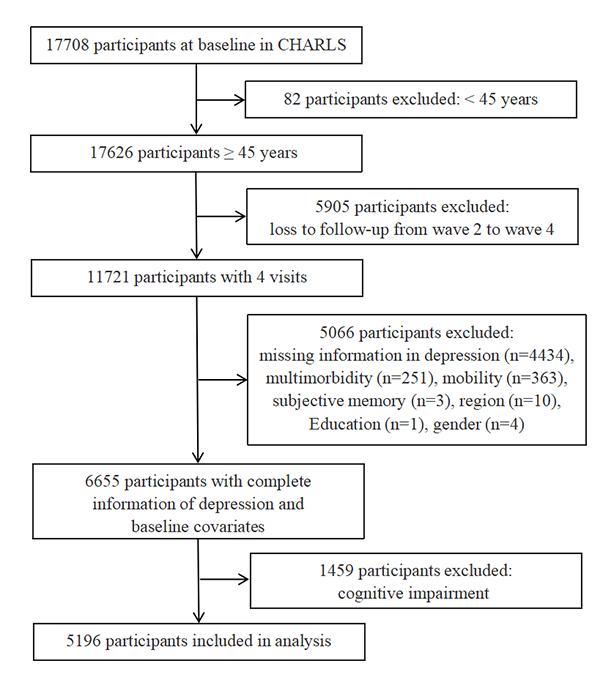


Figure S1. Flow chart of the study population selection

Supplement: Supplementary file 2 — Additional file 2: Fig. S1. Flow chart of the study population selection. [file 12877_2023_3733_MOESM2_ESM.docx]
